# Effect of clozapine and olanzapine on the production of malondialdehyde in reaction to free radical attack on deoxyribose

**DOI:** 10.1192/j.eurpsy.2025.2099

**Published:** 2025-08-26

**Authors:** K. M. Sipowicz, T. B. Pietras, M. K. Kosmalski

**Affiliations:** 1 Department of Interdisciplinary Research in the area of Social Inclusion, The Maria Grzegorzewska University in Warsaw, Warsaw; 2 Department of Clinical Pharmacology, Medical University of Lodz, Lodz, Poland

## Abstract

**Introduction:**

Clozapine and olanzapine are among the most effective antipsychotic drugs. However, their use is associated with the development of metabolic syndrome and lipid profile disorders. It is therefore interesting whether they can affect the level of oxidative stress associated with the Fenton reaction, which is a source of harmful hydroxyl radicals in the blood that damage DNA.

**Objectives:**

The aim of our study was to test the in vitro antioxidant properties of two known neuroleptics – clozapine and olanzapine, which are commonly used in the treatment of schizophrenia and bipolar disorder.

**Methods:**

The study was based on the ability of the hydroxyl radical to split deoxyribose into malondialdehyde (MDA). In the experimental system, the Fenton reaction was used as a source of the hydroxyl radical, in which the divalent iron cation reacts with hydrogen peroxide to form a highly toxic hydroxyl radical. For this purpose, deoxyribose was incubated under appropriate conditions with FeSO4 (0.5 mM), EDTA (1 mM), H2O2 (14 mM) and cloazpine or olanzapine at concentrations of 1, 5, 20 or 50 μmol/l. These concentrations corresponded to the concentration of drugs in the cerebrospinal fluid. A clean system (containing no drugs) was used as a positive control. Then, thiobarbituric acid (TBA) was added to the reaction mixtures in the presence of trichloroacetic acid.

**Results:**

Both olanzapine and clozapine inhibited the formation of malondialdehyde (MDA) from deoxyribose under the influence of the Fenton reaction. At concentrations of 1 and 5 μmol/l, both neuroleptics did not inhibit the reaction. At concentrations of 20 μmol/l, olanzapine inhibited the reaction by 15%, and clozapine by 20%. At concentrations of 50 μmol/l, olanzapine inhibited the reaction by 30%, and clozapine by 37%. The difference between the two neuroleptics was not statistically significant.

**Conclusions:**

1. At concentrations of 1 and 5 umol/l, both neuroleptics did not inhibit the studied reaction.
2. At concentrations of 20 and 50 umol/l, both neuroleptics inhibited the reaction.
3. The difference in the degree of inhibition of the reaction between clozapine and olanzapine was not statistically significant.

The similar results of inhibition of the reaction by both neuroleptics probably result from a similar chemical structure. The fact that clozapine and olanzapine inhibit the Fenton reaction may have a beneficial effect in protecting tissues from oxidative damage.

**Disclosure of Interest:**

None Declared

